# The Present Modes of Treating Consumptives and Their State Control

**Published:** 1897-11

**Authors:** 


The Present Modes of Treating Consumptives and their State Control.—Prof. E. V. Leyden, of Berlin, said that he had no apology to offer for selecting such a well-worn subject, for the misery in the world caused by pulmonary tuberculosis is so great that until we have solved the problem we can never tire of discussing the means of its prevention. He proposed to discuss especially the institutional treatment of consumption and see in what way its benefits may be extended to the poor, who at present are almost wholly debarred from participating in them.

The ancient belief in the incurability of pulmonary tuberculosis was thrown down by Brehmer in 1855, when he established his famous sanitarium in Gorbersdorf and began to restore to society pulmonary invalids who had been condemned to death by physicians and friends. The elements of this method of cure are: the choice of a healthful climate, abundant nourishment, fresh air, hardening of the body and systematic exercise, rest and passive movements, psycho-therapy and discipline of the patient, and lastly, the use of drugs in certain cases.

But before considering these points in detail the speaker would dwell briefly on the prophylaxis of tuberculosis. Assuming as proven that tuberculosis is a contagious disease, and that the agent of infection is Koch's bacillus, he said that contagion may be direct, indirect, or from animals. Direct contagion is usually easily followed, but the path of indirect contagion by means of dust, infected vessels, clothing, or other objects of various kinds is often most obscure. Infection from animals through milk, butter, and meat is, he held, much more frequent than physicians and the public are apt to believe. He laid but little stress on heredity, for he believed that in most cases the disease was transmitted to children by contagion from their parents, and not by inheritance. Our preventive measures are conducted along two lines; the prevention of infection, and the strengthening of the body to resist the attacks of the pathogenic agent. The latter can be effected by bringing up children with Spartan severity and by shunning every approach to coddling. It is along this path that we may hope for success, for the attempt to avoid the agent of contagion must necessarily fail and will result only in causing a bacillophobia that is unworthy of a rational people. The public is justly suspicious of attempts of the sanitary authorities to suppress this disease. Certain precautionary measures, such as the destruction of sputa and the avoidance of intimate association of the sick with the well, are, of course, necessary, but they should be carried out otherwise than by the power of the police. In this connection the speaker took up the question of the alleged danger of sanatoria for consumptives, and denied, with emphasis, that any such existed in a properly conducted institution.

The medicinal treatment, he said, may be considered under three heads, viz., pharmacological products, opotherapy, and orrhotherapy. While admitting the efficacy cf certain drugs of the creosote order, he denied that they had any specific action. Opotherapy with "pulmonin" he dismissed with a word of contempt. He then took up the history of tuberculin and other serum products, and said that they are all as yet only on trial and their actual therapeutic value remains to be proven. There is, however, a very present danger that, in looking forward to ultimate success by this means, we come to value less highly the very efficacious means we have at hand in the institutional treatment of consumption, that is, the hygienic and dietetic therapy of the disease.

Taking up the several points in this treatment, he spoke first of climate. Great faith has for a long time been placed upon a change of climate for the phthisical, especially a change to an elevated region where consumption is seldom encountered among the natives. While valuing highly the climatic advantages of high altitudes, the speaker warned his hearers that there is no actual immunity against tuberculosis in these regions, and we cannot depend upon any climate as in itself directly curative of this disease. Neither are the hills the only regions where benefit may be obtained, for the seashore and southern climates are equally efficacious in many cases. The chief disadvantage of a warm climate is that it unfits the sufferer for a life in his northern home after the cure of his tuberculosis. The speaker quoted Dr. Knopf, of New York, who disbelieves in the specific action of any particular climate and recommends that sanatoria be established within easy distance of the large centres of population.

The assumed specific action of fresh air in tuberculosis is also one of the myths of medicine. It has no such action, but its value is a purely hygienic one, since it aids greatly in strengthening the organism and rendering it more resistant to the attacks of the pathogenic organisms. The air should be pure, as free as possible from dust, not liable to great and sudden changes of temperature, and the place should be free from violent storms.

Of great importance is the nourishment of the phthisical. The time has long passed when men treated consumption as they did other pyretic affections, by a low diet. Experience has shown that the more food the consumptive takes and digests the better are his chances of recovery. Formerly great stress was laid upon the consumption of large quanties of fats, cod-liver oil, cream, and the like, and also upon the taking of a plentiful supply of alcohol. The latter is now given in much smaller quantity, for it is neither a suitable food nor a destroyer of the pathogenic germ. It is useful to cheer the patient and to stimulate his appetite, but in no other way, and it may be harmful by favoring hæmoptysis. We do not even place as much reliance on fats and milk as in former times, but we accommodate the diet to the needs and the taste of the patient, taking care only to see that it is so composed as to furnish calories enough to more than compensate for the daily loss.

The value of psychotherapy in tuberculosis must not be underestimated. The patient should be encouraged to hope for a cure, and should be taken into the physician's confidence to the extent that he may understand the object of each of the measures adopted, so that he may be able to further their good effect by his own co-operation, and may be inspired with confidence in the methods employed. The discipline of the institution, however, should not be too strict, but the patients should be allowed all liberty that is consistent with the proper execution of the hygienic and dietetic rules.

Finally, v. Leyden said, we have to consider the hardening process, and he had reserved the discussion of this for the last in order to emphasize its importance. This is the point in the hygienic and dietetic method which, in a measure, marks its superiority over the specific treatment. The latter combats the pathogenic agents and its success is assured only when it has completely and permanently destroyed the tubercle bacilli so as to secure a lasting immunity for the organism. The hardening process, however, so strengthens the diseased body that it acquires sufficient resisting power not only to tolerate the disease but to overpower it, and so gain a complete and enduring victory over it. Among the measures by which this is effected are a life in the open air, living in airy apartments, and sleeping with open windows. Hydrotherapy is another powerful agent in this process, and consists in cold sponging, showers, and short cold plunges. Further, exercises in the open air are necessary, such as methodically prescribed hill climbing, etc.

